# Factors Associated With Not Smoking and Ceasing Smoking During Pregnancy Among Aboriginal and Torres Strait Islander Women

**DOI:** 10.1093/ntr/ntaf126

**Published:** 2025-06-17

**Authors:** Jamie Bryant, Rita Hitching, Matthew Clapham, Sandra Eades, Kristy Fakes, Bob Davis, Jennifer Rumbel, Emilie Cameron

**Affiliations:** School of Medicine and Public Health, College of Health, Medicine and Wellbeing, University of Newcastle, Callaghan, Australia; Equity in Health and Wellbeing Program, Hunter Medical Research Institute, New Lambton Heights, Australia; School of Medicine and Public Health, College of Health, Medicine and Wellbeing, University of Newcastle, Callaghan, Australia; Equity in Health and Wellbeing Program, Hunter Medical Research Institute, New Lambton Heights, Australia; Clinical Research Design and Statistics Support Unit, Hunter Medical Research Institute, New Lambton Heights, Australia; Centre for Epidemiology and Biostatistics, School of Population and Global Health, University of Melbourne, Carlton, Australia; School of Medicine and Public Health, College of Health, Medicine and Wellbeing, University of Newcastle, Callaghan, Australia; Equity in Health and Wellbeing Program, Hunter Medical Research Institute, New Lambton Heights, Australia; School of Medicine and Public Health, College of Health, Medicine and Wellbeing, University of Newcastle, Callaghan, Australia; Equity in Health and Wellbeing Program, Hunter Medical Research Institute, New Lambton Heights, Australia; Systems Neuroscience Group, School of Psychological Sciences, Hunter Medical Research Institute, New Lambton Heights, Australia; School of Medicine and Public Health, College of Health, Medicine and Wellbeing, University of Newcastle, Callaghan, Australia; Equity in Health and Wellbeing Program, Hunter Medical Research Institute, New Lambton Heights, Australia

## Abstract

**Introduction:**

Smoking during pregnancy has been linked to serious adverse health outcomes for mother and baby.

**Aims and Methods:**

This study aims to examine the individual and pregnancy characteristics associated with not smoking or ceasing smoking during pregnancy among Aboriginal and Torres Strait Islander women in Australia. A cross-sectional secondary analysis of data was conducted. Records were included if the mother was Aboriginal and/or Torres Strait Islander and had smoking status recorded. The individual and pregnancy characteristics associated with not smoking or ceasing smoking during pregnancy were assessed using mixed model logistic regressions.

**Results:**

A total of 49 651 women with a median age of 25 years were included. Overall, 56% (*n* = 27 782) reported not smoking during pregnancy and 13% reported ceasing smoking during pregnancy. The odds of not smoking were increased for those who were older, had fewer previous pregnancies, were married or in a de facto relationship, were private patients, had higher body mass index, were diagnosed with hypertension, attended more antenatal care visits, and began antenatal care earlier in pregnancy. The odds of ceasing smoking were higher for those who were married or in a de facto relationship, were private patients, began antenatal care earlier in pregnancy, had a lower number of pregnancies, and were diagnosed with hypertension.

**Conclusions:**

A variety of sociodemographic, health, and antenatal care factors were associated with smoke-free pregnancies among Aboriginal and Torres Strait Islander women. These findings inform targeted interventions and policies to enhance efforts to promote smoke-free pregnancies among Aboriginal and Torres Strait Islander women.

**Implications:**

Understanding the factors associated with not smoking among pregnant Aboriginal and Torres Strait Islander woman can inform the development of strategies to address the harmful effects of smoking during pregnancy. This secondary analysis found that considering strategies such as tailoring supports to those who are younger or not married, evaluating programs run by different jurisdictions, and addressing barriers to receiving early and consistent antenatal care may be useful for informing the development of targeted interventions and policies and tailoring cessation support approaches.

## Introduction

Smoking during pregnancy has been causally linked to serious adverse health outcomes during the gestational period for both mother and baby. For mothers, these outcomes include pre-eclampsia, placenta previa, hyperemesis gravidarum,^1^ and for babies, these adverse outcomes include intrauterine growth restriction and stillbirth.^2^^,^^3^ Higher rates of pregnancy-related morbidity and mortality associated with smoking during pregnancy, and to a lesser extent, second-hand smoke,^4^ also extend into the perinatal and neonatal periods, and into childhood and beyond. Smoking during pregnancy has been found to have both short and long-term consequences, including prematurity, low birth weight, increased risk of sudden infant death syndrome, increased risk of obesity, asthma, and other respiratory diseases in childhood, impaired neurodevelopment, and increased risk of some cancers.^2^^,^^3^^,^^5^ Ceasing smoking during pregnancy, however, reduces the risk of adverse pregnancy outcomes and longer-term health impacts to levels almost the same as non-smokers.^6–8^

In Australia, Aboriginal and Torres Strait Islander communities experience high rates of maternal and infant morbidity and mortality,^9^ partly as a result of high rates of smoking during pregnancy.^10^ High rates of smoking reflect ongoing and complex impacts of colonization, as well as the direct impact of the Tobacco Industry, which continues to actively target Aboriginal and Torres Strait Islander people.^11^ As a result of Aboriginal and Torres Strait Islander community leadership, there has been significant investment over the past decade in reducing smoking uptake and promoting smoking cessation among pregnant Aboriginal and Torres Strait Islander women. Through this leadership, the proportion of Aboriginal and Torres Strait Islander pregnant women who report smoking at any time during pregnancy has decreased from 50% to 40% in 2021.^9^ However, the goal set in the Aboriginal and Torres Strait Islander Health Plan 2013–2023 to reduce the prevalence of smoking to 37% by 2023 was not met^12^ and addressing smoking during pregnancy remains a key national priority. The updated National Tobacco Strategy 2023–2030^13^ explicitly recommends expanding efforts to prevent and reduce tobacco use among Aboriginal and Torres Strait Islander people, with pregnant women identified as a specific priority group.

Understanding the factors associated with not smoking during pregnancy and the factors associated with smoking cessation during pregnancy is critical to the continued development of effective interventions and population health approaches that improve maternal and infant health outcomes. A systematic review and meta-analysis of 54 studies (including 15 randomized controlled trials) globally identified a range of sociodemographic, psychosocial, and pregnancy characteristics associated with stopping smoking during pregnancy.^14^ While a small cross-sectional study has explored factors associated with smoke-free pregnancies among Aboriginal and Torres Strait Islander women,^15^ no studies have explored the factors associated with not smoking and successful smoking cessation during pregnancy, among Aboriginal and Torres Strait Islander women during pregnancy at a population level. Consequently, this study aims to describe the sociodemographic and pregnancy characteristics associated with (1) not smoking during pregnancy and (2) ceasing smoking during pregnancy, among Aboriginal and Torres Strait Islander women in Australia for the period of 2014–2017.

## Materials and Methods

### Aboriginal and Torres Strait Islander Governance and Research Team Positionality

The research team recognizes that their lived experience and worldviews influence the way that the study was conducted, and results were interpreted. The study was conceptualized by SE (Noongar) and JB (non-Indigenous) as part of a National Health and Medical Research Centre for Research Excellence Grant focused on Aboriginal Child and Adolescent Health. Our team brings together Aboriginal and Torres Strait Islander lived experience (SE, BD (Dungutti/Biripi), JR (Gamilaraay), expertise in Aboriginal health services research (SE, BD, JR, JB), and health behavior scientists (JB, EC, KF, RH). An Aboriginal and Torres Strait Islander Reference Group, consisting of five Aboriginal members with expertise in Aboriginal health research, delivery, policy development, and evaluation (SE, BD, JR, Nicole Turner [Kamilaroi] and Steve Blunden [Dunghutti]), was established to oversee the research process and interpretation of results. The Reference Group ensured that the study was conducted in accordance with core values for ethical research with Aboriginal and Torres Strait Islander Peoples.

### Study Design

Secondary analysis of data from the National Perinatal Data Collection for the period 2014–2017. The National Perinatal Data Collection is a national collection of pregnancy and childbirth data consisting of an agreed set of standardized data items, as specified in the Perinatal National Minimum Dataset, as well as additional items. Data are sourced from notification forms filled out for each birth by midwives and other staff, using information obtained from mothers and from hospital or other records. An extract from each jurisdictional data collection is supplied annually to the Australian Institute of Health and Welfare to form the National Perinatal Data Collection.^16^ Data were accessed using the Sax Institute Secure Unified Research Environment from March 2022.

### Study Sample

Data were available for all births recorded in the National Perinatal Data Collection from January 1, 2014 to December 31, 2017 with Indigenous status and smoking status recorded. The data used in this study included all births where the mother was an Aboriginal and/or Torres Strait Islander woman aged 18 years and over.

### Measures

A detailed explanation of each variable is provided in File S1.

#### Smoking Status

The National Perinatal Data Collection contains four self-reported smoking variables: smoking in first 20 weeks of pregnancy (no, yes, not recorded); smoking after 20 weeks of pregnancy (no, yes, not recorded); average number of cigarettes smoked per day during first 20 weeks of pregnancy; average number of cigarettes smoked per day after 20 weeks of pregnancy. Women were classified as not smoking if they reported not smoking during the first 20 weeks of pregnancy. Smoking cessation was defined as those who reported smoking during the first 20 weeks of pregnancy but not after 20 weeks of pregnancy. A small percentage (1.1%; *n* = 548) of women reported smoking after 20 weeks of pregnancy and not in the first 20 weeks and were not included in smoking counts, in line with standard reporting.^17^^,^^18^

#### Individual Level Factors

Data were obtained for each woman on state/territory where the birth took place; maternal age in years; pre-pregnancy body mass index (BMI); and marital status (married or de facto, never married, widowed, divorced or separated, not recorded). Data were also obtained on insurance classification, the mother was admitted to hospital of birth under (public, private, not applicable, eg, home birth) as a proxy for socioeconomic position, reflecting that access to private healthcare services in Australia is associated with socioeconomic position.^19^

#### Pregnancy Characteristics

For each birth data were provided on: year of baby’s birth (2014–2017); Number of antenatal care visits; gestational age in completed weeks at first antenatal visit; parity (total number of previous pregnancies resulting in a live birth or still birth); diabetes status during pregnancy (diabetes, no diabetes or not stated, not recorded); and Hypertension status during pregnancy (hypertension, no hypertension or not stated, not recorded). To maintain the privacy of individuals in the dataset, some continuous variables were grouped and categorized at the extreme ends of their range. This included those 45 years and over coded as 45; those 19 years and under coded as 19; those with a BMI under 14 kg/m^2^ coded as 13; those with a BMI over 55 kg/m^2^ coded as 55; those with parity of 5 and over coded as 5; and those with 20 and over antenatal care visits coded as 20. These accounted for at most 10% of the sample.

### Statistical Analysis

Analyses were conducted in R version 4.0.3 (2020-10-10). Individual-level factors and pregnancy characteristics are reported as counts and proportions for categorical variables and median and interquartile range (IQR) for continuous variables. The association of not smoking during pregnancy with individual level factors and pregnancy characteristics was assessed with mixed model logistic regressions. Univariate and multivariate regressions were fitted separately with the main outcome “not smoking during the first 20 weeks of pregnancy”. For the regressions, the factors maternal age, BMI, number of antenatal visits, and gestational age at first antenatal visit were modeled using natural cubic splines with knots at 16.6%, 33.3%, 50%, 66%, and 83.3% percentiles. Odds ratios are presented as the ratio of odds at 75th and 50th percentiles compared to the odds at 25th percentile. Percentiles were based on all births in the National Perinatal Data Collection for 2014–2017. Parity was modeled as a linear trend. The categorical factors marital status, patient insurance classification, diabetes status, hypertension status, year of baby’s birth, and Australian State/Territory where the birth took place were also included in the regressions. Univariate and multivariate regressions were fitted in the same way for the main outcome “ceasing smoking during pregnancy”. Files S2 and S3 have plots of the predicted probability of not smoking as a function of each continuous characteristic, estimated with other variables set to the median or reference category as appropriate.

The regression modeling was conducted as a complete case analysis with missing data treated as missing at random. Five variables had data missing for over 15% of the sample. This was due to administrative differences, which meant some variables were not reported for some states or other jurisdictions over some years. BMI was not reported in New South Wales in 2014–2015. The number of antenatal visits was not reported for Victoria in 2014–2015, marital status was not reported in Western Australia in any year, and diabetes and hypertension status were not reported in Victoria in any year. To examine the effect of the missing data, a sensitivity analysis was conducted by repeating the multivariate regressions using multiple imputation for the missing values. This was conducted using chained equations (MICE)^20^ and Rubin’s rules^21^ to pool the imputed regression results. Due to the size of the data set, five imputations were used with predictive mean matching estimation method.

## Results

### Sample Description

There was a total of *n* = 1 203 717 births to women aged 18 and over in Australia between 2014 and 2017 with valid Indigenous status and smoking status in the first 20 weeks of pregnancy recorded. Of these 4.1% (*n* = 49 651) were births to Aboriginal and/or Torres Strait Islander women and included in this study. The demographics and pregnancy characteristics of the sample are shown in Table 1. The median age of the women was 22 years (IQR 22–30). Half (*n* = 21 238) were in a married or de facto relationship and 96% (*n* = 47 345) were classified as a public patient for the birth. Overall, 56% (*n* = 27 782) were recorded as not smoking during the first 20 weeks of pregnancy and 13% ceased smoking during pregnancy.

**Table 1.** Demographic and Pregnancy Characteristics for Aboriginal and/or Torres Strait Islander Pregnant Women by Smoking Status (*n* = 49 651)

Variable	Characteristic	No smoking^1^		Smoking^2^	Ceased smoking^3^
		*N* (%)		*N* (%)	*N* (%)
Total		27 782 (56%)		21 869 (44%)	2742 (13%)
Year	2014	6524 (55%)		5389 (45%)	672 (13%)
	2015	6726 (55%)		5430 (45%)	736 (14%)
	2016	7254 (57%)		5506 (43%)	680 (13%)
	2017	7278 (57%)		5544 (43%)	654 (12%)
	Missing	0		0	0
State/Territory	NSW	8890 (59%)		6204 (41%)	1079 (17%)
	Vic	2116 (57%)		1597 (43%)	199 (14%)
	Qld	8593 (56%)		6659 (44%)	705 (11%)
	SA	1407 (51%)		1350 (49%)	178 (15%)
	WA	3428 (52%)		3133 (48%)	275 (8.8%)
	Tas	656 (63%)		390 (37%)	30 (7.7%)
	NT	2395 (51%)		2316 (49%)	253 (11%)
	ACT	297 (57%)		220 (43%)	23 (10%)
	Missing	0		0	0
Patient insurance classification	Public patient	25 863 (55%)		21 482 (45%)	2674 (13%)
	Private patient	1796 (86%)		294 (14%)	56 (20%)
	Missing or not applicable	123		93	12
Diabetes status	Diabetes during pregnancy	3668 (60%)		2472 (40%)	329 (14%)
	No diabetes/ not stated	21 998 (55%)		17 800 (45%)	2214 (13%)
	Missing	2116		1597	199
Hypertension	Hypertension during pregnancy	1951 (66%)		1027 (34%)	175 (18%)
	No hypertension/not stated	23 715 (55%)		19 245 (45%)	2368 (12%)
	Missing	2116		1597	199
Marital status	Married or de facto	13 704 (65%)		7534 (35%)	1049 (14%)
	Never married	9931 (48%)		10 571 (52%)	1338 (13%)
	Widowed, divorced, separated	445 (48%)		491 (52%)	60 (13%)
	Missing	3702		3273	295
		Median (IQR)		Median (IQR)	Median (IQR)
Maternal age	Years	25 (22, 30)		25 (22, 30)	25 (21, 29)
	Missing	0		0	0
Pre-pregnancy BMI	kg/m^2^	26.6 (22.4, 32.0)		25.2 (21.3, 30.2)	25.7 (21.6, 30.9)
	Missing	5527		4757	659
Number of Antenatal care visits	Number	10 (7, 11)		8 (5, 10)	10 (7, 11)
	Missing	1322		1201	136
Gestational age at the first antenatal care visit	Weeks	11 (7, 16)		12 (8, 19)	11 (7, 17)
	Missing	554		805	46
Parity	Number	1 (0, 2)		2 (1, 3)	1 (0, 2)
	Missing	53		53	4
Av. number of cigarettes smoked per day during first 20 weeks of pregnancy	Number	0 (0, 0)		7 (5, 10)	5 (3, 10)
	Missing	0		6086	776
Av. number of cigarettes smoked per day after first 20 weeks of pregnancy	Number	0 (0, 0)		5 (3, 10)	0 (0, 0)
	Missing	146		1244	0

^1^No smoking self-reported in the first 20 weeks of pregnancy. Row total of no smoking and smoking are the denominators for percentages.

^2^Smoking self-reported in the first 20 weeks of pregnancy. Row total of no smoking and smoking are the denominators for percentages.

^3^Smoking self-reported in the first 20 weeks of pregnancy and not after 20 weeks. Denominator for ceased smoking percentage is number smoking in first 20 weeks of pregnancy with smoking status after 20 weeks recorded (*n* = 21 325).

### Factors Associated with Not Smoking

**Figure 1.**
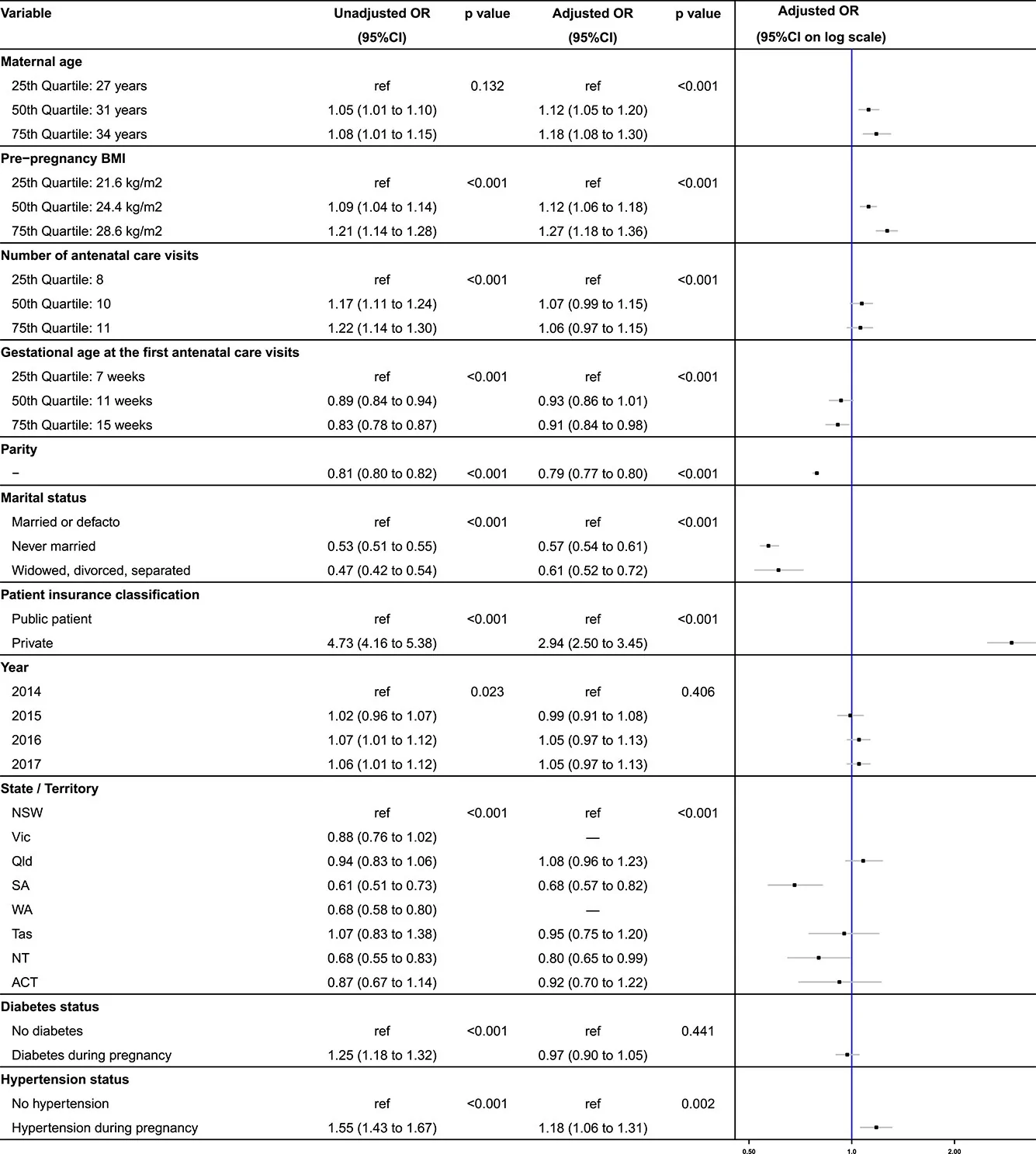
Forest plot of the factors associated with not-smoking during pregnancy for Aboriginal and/or Torres Strait Islander women (*n* = 29 260 for adjusted model).

Figure 1 presents the univariate and multivariate regressions for factors associated with not smoking in the first 20 weeks of pregnancy for Aboriginal and/or Torres Strait Islander women. Accounting for other factors, the odds of not smoking increased with maternal age and BMI and decreased with higher gestational age at the first antenatal care visit and parity (all *p* < .001). For the number of antenatal visits, the odds of not smoking increased with the number of visits (*p* < .001) until around 10 visits (in File S2). Those who were married or in a de facto relationship had greater odds of not smoking than those who were never married or were widowed, divorced or separated (65% not smoking vs. 48%, *p* < .001) and those classified as private patients at the time of birth were more likely to not smoke than those classified as public patients (86% vs. 55%, *p* < .001). The odds of not smoking were higher in those diagnosed with hypertension during pregnancy than those without hypertension (66% vs. 55%, *p* = .002). There was no association between diabetes status and smoking in the adjusted model (60% vs. 55%, *p* = .441). There was some variation between states and territories (*p* < .001) with the odds of not smoking lower in South Australia (51%), Western Australia (52%), and Northern Territory (51%) compared to NSW (59%). There was no difference between the years included in the study (*p* = .406). Conclusions were not affected by missing values.

### Factors Associated with Ceasing Smoking

**Figure 2.**
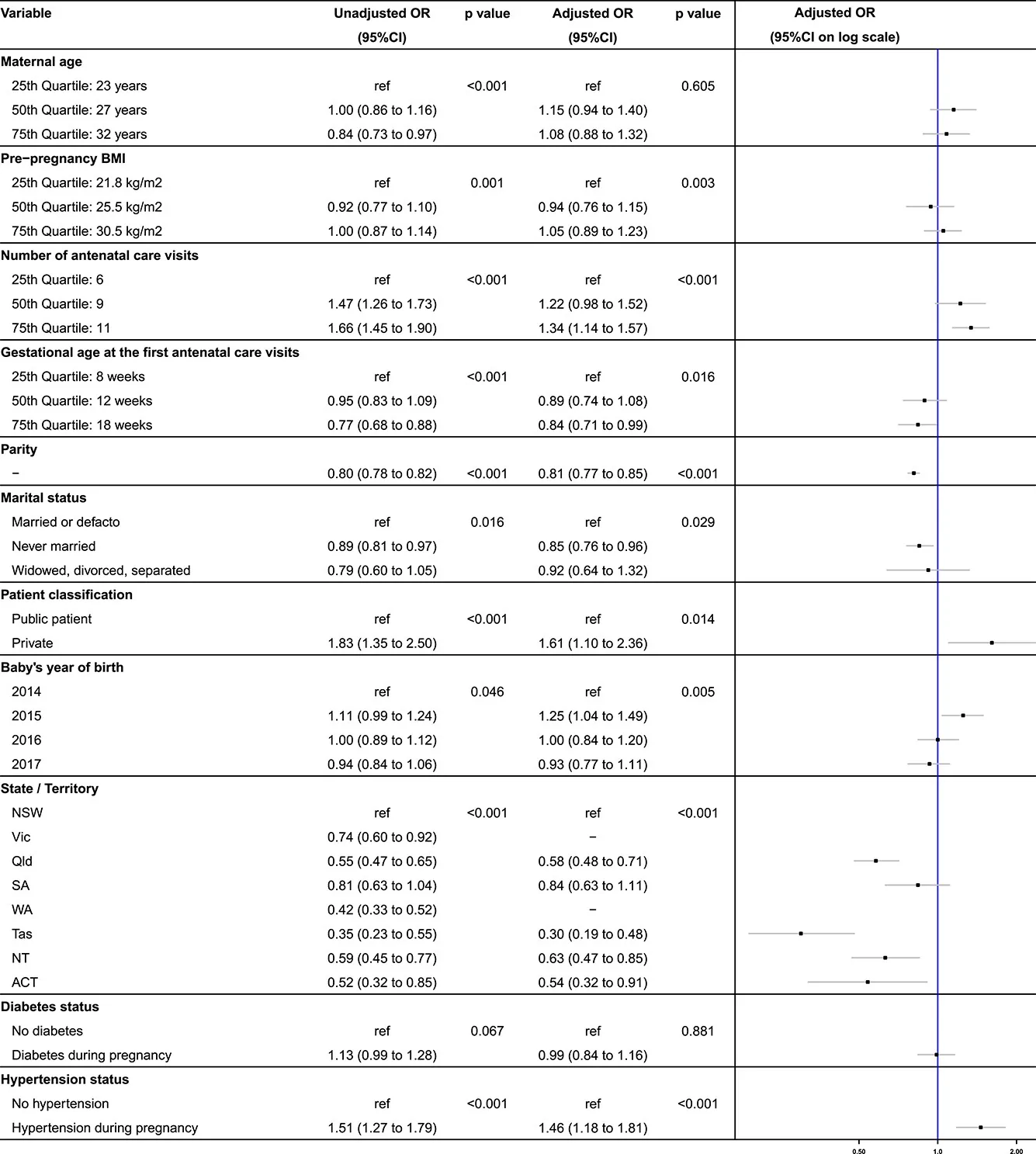
Forest plot of the factors associated with ceasing smoking during pregnancy for Aboriginal and/or Torres Strait Islander women (*n* = 12 308 for adjusted model).

The univariate and multivariate regressions for factors associated with ceasing smoking during pregnancy among Aboriginal and/or Torres Strait Islander women are presented in Figure 2. These conclusions were not impacted by missing values investigated through sensitivity analysis. Accounting for other factors, those with increased odds of ceasing smoking were married or de facto compared to never married (14% vs. 13%, *p* = .029), had a private patient classification compared to being a public patient for the birth (20% vs. 13%, *p* < .014), had the first antenatal visit at a younger gestational age (*p* < .016) and had a lower number of previous pregnancies (*p* < .001). The trend associated with BMI was typically increasing probability with increasing BMI (*p* = .003); however, it was flat or not statistically significant between the first and third quartile of BMI, where approximately 50% of participants fell (in File S3). Similarly, the relationship between ceasing smoking and antenatal visits increased (*p* < .001) to about 10 visits and then started to decrease. Those diagnosed with hypertension during pregnancy had increased odds of not smoking compared to those not diagnosed (18% vs. 12%, *p* < .001); however, this was not the case for those diagnosed with diabetes (14% vs. 13%, *p* = .881). Cessation rates varied between states (*p* < .001), with NSW (17%), SA (15%), and VIC (14%) having the highest rates. There was no association between ceasing smoking and mother’s age when adjusting for other factors (*p* = .605).

## Discussion

This study aimed to describe the sociodemographic and pregnancy characteristics associated with not smoking during the first 20 weeks of pregnancy and ceasing smoking after 20 weeks of pregnancy among Aboriginal and Torres Strait Islander women using routinely collected data from National Perinatal Data Collection. Findings highlight the complex interplay of sociodemographic, pregnancy, and biomedical factors in shaping smoking and quitting behaviors among Aboriginal and Torres Strait Islander women in Australia.

Highlighting the potential role of socioeconomic status, social support and access to healthcare resources in promoting smoke-free pregnancies for Aboriginal and Torres Strait Islander women, those who were married or in a de facto relationship and those admitted as a private patient for the birth were more likely to not smoke and cease smoking than those who were never married or widowed and those admitted as a public patient, respectively. Evidence shows that social support and access to advice during a quit attempt facilitate smoking cessation among Aboriginal and Torres Strait Islander women.^22^ Behavior changes, either before falling pregnant or early in pregnancy, may also be prompted by health professionals and trusted family and friends discussing the harmful effects of smoking during pregnancy for both mother and baby.^22^

Positive associations were found between maternal age and the likelihood of not smoking during the first half of pregnancy. This aligns with previous research indicating that older mothers may be more motivated to adopt healthier behaviors during pregnancy.^23–26^ There is limited information about the impact of age on not smoking during pregnancy for Aboriginal and Torres Strait Islander women. The only study to examine this found that although not statistically significant, participants in the older age groups appeared to have a higher chance of staying smoke-free during pregnancies compared to participants aged between 16 and 25 years.^15^^,^^27^

Study findings provide important information about disparities in smoking and quitting behaviors across different states and territories, with implications for targeted interventions and policy initiatives. The odds of not smoking in the first half of pregnancy were lower in South Australia, Western Australia, and the Northern Territory, which are also three of the four jurisdictions with the highest rates of smoking in the general Aboriginal and Torres Strait Islander population.^28^ This finding underscores the need for region-specific strategies to address local barriers and challenges. While there has been significant investment in Indigenous smoking cessation and in particular, programs for pregnant women (eg, Quit for New Life in NSW^18^), geographical variation highlights the need for continual adaptation and evaluation of smoking cessation strategies, accounting for factors such as accessibility, socioeconomic conditions, and cultural attitudes towards smoking.^22^

The impact of health and biomedical factors on not smoking and quitting smoking during pregnancy was variable. Women with higher BMI had higher odds of not smoking and of ceasing smoking. This finding aligns with a number of other studies that show that individuals who smoke generally have a lower BMI,^29^^,^^30^ a correlation that is often attributed to the appetite-suppressing effects of nicotine on body metabolism. While hypertension was associated with increased odds of not smoking during pregnancy and ceasing smoking, no significant associations were found for the impact of diabetes. The diagnosis of hypertension, including both chronic hypertension and gestational hypertension, may act as a strong motivator for women to cease smoking. Chronic hypertension, in particular, may lead to heightened awareness and interventions by healthcare providers, who can counsel women on the importance of smoking cessation to mitigate risks to both maternal and fetal health in line with clinical practice guidelines.^31^^,^^32^ Failure to find any associations between smoke-free pregnancies and diabetes may be influenced by the type of diabetes considered. Chronic diabetes can have long-term health effects that may motivate women to quit smoking, while gestational diabetes that develops during pregnancy likely arises too late to influence smoking behavior as measured in this dataset. The varying levels of healthcare interventions received by women with both chronic and gestational diabetes, along with the relationship between diabetes, maternal age, and BMI,^33^ might also explain the observed lack of association between diabetes and smoking cessation in pregnancy. These findings underscore the importance of integrating lifestyle management into prenatal care, offering a holistic approach to improving maternal health outcomes.

The significance of early and consistent prenatal care in supporting smoking cessation efforts was shown in the finding that beginning antenatal care earlier in pregnancy and having more antenatal visits were associated with increased odds of not smoking or ceasing smoking during pregnancy. Current Australian Pregnancy Care Guidelines^34^ recommend assessment of the mother’s smoking status and exposure to passive smoking at the first antenatal appointment, ideally early in the first trimester. Because pregnancy often serves as a catalyst for women who smoke to consider quitting, these guidelines encourage clinicians to provide the mother (and her partner) with information about the risks associated with smoking during pregnancy and information on the benefits of smoking cessation. However, these recommendations rely not only on Western values and perspectives of health, risk, and safety but also on women accessing antenatal care early in their pregnancy. Aboriginal and Torres Strait Islander women are not only 7% less likely than non-indigenous women to attend an antenatal visit in the first trimester, they are 7% more likely to receive less than five antenatal appointments, half of the 8–10 visits recommended for uncomplicated pregnancies.^9^ This disparity underscores the critical need to continue to address the barriers that Aboriginal and Torres Strait Islander women face in accessing early and consistent prenatal care, including well-known systemic issues, including geographic isolation, racism, and mistrust of healthcare systems.^35^^,^^36^

### Strengths and Limitations

This study took a strengths-based approach by investigating protective factors associated with not smoking that can be built upon when developing policies and initiatives. The establishment of a comprehensive Aboriginal and Torres Strait Islander governance group that oversaw the research process and interpretation of results was a strength of the study, and provided critical feedback to ensure the interpretation of results was grounded in the historic and contemporary lived experiences and needs of Aboriginal and Torres Strait Islander children and families. The study used routinely collected data recorded for all births in Australia. As such, our analysis was limited to factors included in the dataset and other important factors that might affect the outcomes (such as psychosocial factors) could not be included. Additionally, the dataset relies on women accurately responding to questions about smoking and could therefore underestimate the true smoking rate.^37^^,^^38^ It also relies on correct recording of Aboriginal and Torres Strait Islander identity in the national dataset, and studies in Australia linking perinatal data with birth registration data and hospital admissions show that Aboriginal and Torres Strait Islander status is under-recorded.^39^ This means that the actual number of Aboriginal and Torres Strait Islander individuals may be underestimated in this dataset.^40^ Based on the data available, the study defines cessation only as reporting smoking at any time during the first 20 weeks of pregnancy and not at all after the first 20 weeks. It does not consider those who quit later in pregnancy or who had periods of not smoking. Women who had multiple births in the study timeframe were captured multiple times in the dataset. Given that those with fewer previous births are more likely not to smoke, this could lead to an underestimation of non-smoking. However, this is likely to have only a very small or negligible impact on the results. Due to differences across jurisdictions, there was a large amount of missing data in the demographic and pregnancy factors. The effect of the missing data was investigated in a sensitivity analysis and was found not to impact the conclusions. Finally, only women aged 18 years and older were included in the analysis, as the dataset focused on adult populations and excluded younger women who may have different smoking behaviors and risk factors. Over the study period, approximately 14.6% of Aboriginal and Torres Strait Islander women aged under 20 years were pregnant (note that national data is not published for age categories <20 years of age). This equates to less than 3000 women excluded from the study due to age.

## Conclusions

Not smoking during pregnancy is critically important for the health and well-being of the mother and baby in both the short and long term. A number of sociodemographic, health, and antenatal care factors were found to be associated with both not smoking during the first half of pregnancy and/or ceasing smoking during pregnancy, among Aboriginal and Torres Strait Islander women. This data provides important information about the characteristics associated with successful smoke-free pregnancies among Aboriginal and Torres Strait Islander women that can be used to inform the development of targeted interventions and policies, tailor cessation support approaches, and enhance public health strategies to effectively address and reduce smoking among Aboriginal and Torres Strait Islander pregnant women.

## Supplementary Material

Supplementary_files_1-3_ntaf126

## Data Availability

Access to the National Perinatal Data Collection used in this study can be arranged through the Australian Institute of Health and Welfare.
