# Supplementary material for: Factors Associated With Not Smoking and Ceasing Smoking During Pregnancy Among Aboriginal and Torres Strait Islander Women
Source: Nicotine Tob Res. 2025 Jun 17;28(10):1650–8. doi: 10.1093/ntr/ntaf126 (PMC13601962; doi:10.1093/ntr/ntaf126)
Supplement: Supplementary file 1 [file supplementary_files_1-3_ntaf126.docx]

**Supplementary Files**

**Supplementary File S1:** Explanation of each variable from National Perinatal Data Collection^1^ used in the analysis.

| **Variable** | **Field name** | **Description** | **Value** | **Meaning** | **Notes** |
| --- | --- | --- | --- | --- | --- |
| Year | YEAR | Year of baby’s birth | 2014-2017 | Year of birth |  |
| indigenous status | IND_MOTH | Maternal Indigenous status | Indigenous | Aboriginal and/or Torres Strait Islander origin | Indigenous status is self-reported and recorded in line with national guidelines^2^. Those with missing value (0-2% across states)  not included in data set. |
|  |  |  | Non-Indigenous | Neither Aboriginal or Torres Strait Islander origin |  |
|  |  |  | missing | Missing or not stated or inadequately described |  |
| State/Territory | STATE | State / territory where the birth took place | NSW | New South Wales |  |
|  |  |  | Vic | Victoria |  |
|  |  |  | Qld | Queensland |  |
|  |  |  | SA | South Australia |  |
|  |  |  | WA | Western Australia |  |
|  |  |  | Tas | Tasmania |  |
|  |  |  | ACT | Australian Capital Territory |  |
|  |  |  | NT | Northern Territory |  |
| Marital status | MAR_STAT | Marital status | Married or de facto |  | Variable not available for WA. |
|  |  |  | Widowed, divorced or separated |  |  |
|  |  |  | Never married |  |  |
|  |  |  | missing |  |  |
| Patient insurance classification | PAT_CLSS | Patient insurance classification at hospital of birth | Public | Admitted as public patient |  |
|  |  |  | Private | Elected to use private health insurance for admission |  |
|  |  |  | Not applicable | Outside hospital system eg. Home birth |  |
|  |  |  | missing | No data provided |  |
| Diabetes | DIABSTAT | Diabetes status during pregnancy | Diabetes during pregnancy | Any diabetes recorded | Data are not available for Vic. |
|  |  |  | No diabetes/ not stated | No diabetes recorded |  |
|  |  |  | missing | no data provided |  |
| Hypertension | HYPTSTAT | Hypertension status during pregnancy | Hypertension during pregnancy | Any hypertension recorded | Data are not available for Vic. |
|  |  |  | No hypertension/ not stated | No hypertension recorded |  |
|  |  |  | missing | No data provided |  |
| Maternal age | MOTH_AGE | Maternal age at birth in years | 19 | 19 years and under (grouped) | Values at ends of range combined to maintain privacy. |
|  |  |  | 20-44 | 20-44 years |  |
|  |  |  | 45 | 45 and over (grouped) |  |
| Pre-pregnancy BMI | BMI | Pre-pregnancy Body Mass Index (BMI) | 14 | Less than 14 kg/m^2^ (grouped) | Values at ends of range combined to maintain privacy. Data quality and definitions used may vary by state/territory and year. Data are not available for BMI for NSW in 2014 or 2015 and are missing 1.6-16% across jurisdictions and years. |
|  |  |  | 14.0-55.0 | 14-55 kg/m^2^ |  |
|  |  |  | 55 | Greater than 55 kg/m^2^ (grouped) |  |
|  |  |  | missing | No data provided |  |
| Antenatal care visits | ANTVISIT | Antenatal visits | 0-19 | 0-19 visits | Values at ends of range combined to maintain privacy. Data are not available for ANTVISIT in Vic until 2016. |
|  |  |  | 20 | Greater than or equal to 20 visits (grouped) |  |
|  |  |  | missing | No data provided |  |
| Gestational age at the first antenatal care visit | FRSTGAGE | Pregnancy duration in completed weeks at first antenatal visit | 0-42 | Gestational age in completed weeks |  |
|  |  |  | missing | No data provided |  |
| Parity | PARITY | Total number of previous pregnancies resulting in a livebirth or a stillbirth | 0-4 | 0-4 previous pregnancies | Values at ends of range combined to maintain privacy. |
|  |  |  | 5 | 5 and over (grouped) previous pregnancies |  |
|  |  |  | missing | No data provided |  |
| SA3 | SA3 | SA3 of usual residence | ABS SA3 codes | Statistical Area 3 (SA3) of usual residence (ASGS 2011). | Data are based on ASGS 2011 for 2014-2016 and ASGS 2016 for 2017. Data for ACT in 2014 and 2015 were provided as SLAs, which can fall across more than one SA3/SA4. SA3/SA4s for these years have been calculated based on a random, weighted approach. |
|  |  |  | missing | No data provided |  |
| SA4 | SA4 | SA4 of usual residence | ABS SA4 codes | Statistical Area 4 (SA4) of usual residence (ASGS). |  |
|  |  |  | missing | No data provided |  |
| Smoking status in first 20 weeks of pregnancy | SMOKSTAT1 | Self-reported smoking in first twenty weeks of pregnancy | Smoked |  | Responded to question: *Did you smoke at all in the first 20 weeks of pregnancy?*  Those with missing value (0-10% across states/territories) were not included in data set |
|  |  |  | Did not smoke |  |  |
|  |  |  | missing | No data provided |  |
| Smoking status after first 20 weeks of pregnancy | SMOKSTAT2 | Self-reported smoking after twenty weeks of pregnancy | Smoked |  | Responded to question asked after birth: *Did you smoke at all after 20 weeks of pregnancy?* |
|  |  |  | Did not smoke |  |  |
|  |  |  | missing | No data provided |  |
| Av. number of cigarettes smoked per day during first 20 weeks of pregnancy | SMOKQUAN1 | Self-reported average number of cigarettes smoked per day during first 20 weeks of pregnancy | 0 | None | Values at ends of range combined. Data are not available for Tas, Vic, SA or NT. |
|  |  |  | 0.5 | Less than 1 per day (occasional) |  |
|  |  |  | 1-30 | 1-30 per day |  |
|  |  |  | 31 | Greater than 30 (grouped) |  |
|  |  |  | missing | No data provided |  |
| Av. number of cigarettes smoked per day after first 20 weeks of pregnancy | SMOKQUAN2 | Self-reported average number of cigarettes smoked per day after 20 weeks of pregnancy | 0 | None | Values at ends of range combined. Data are not available for SMOKQUAN2 for Tas. |
|  |  |  | 0.5 | Less than 1 per day (occasional) |  |
|  |  |  | 1-30 | 1-30 per day |  |
|  |  |  | 31 | Greater than 30 (grouped) |  |
|  |  |  | missing | No data provided |  |

^1^ Australian Institute of Health and Welfare, 2024. National Perinatal Data Collection, 2019: Quality Statement. Exported from METEOR (AIHW’s Metadata Online Registry). Australian Institute of Health and Welfare, Australian Government.

^2^ Australian Institute of Health and Welfare 2010. National best practice guidelines for collecting Indigenous status in health data sets. Cat. no. IHW 29. Canberra: AIHW.

**Supplementary file S2:** Predicted probability of not smoking in first 20 weeks of pregnancy for Aboriginal and/or Torres Strait Islander women. Probability estimates at: marriage=Married or de facto, state=NSW, patient class=Public, year=2014, mothers age=25 years, BMI=26 kg/m^2^, parity=1, antenatal visits=9, gestational age at first visit=12 weeks, diabetes status=No diabetes, hypertension=No hypertension.

**Supplementary File S3:** Predicted probability of ceasing smoking for Aboriginal and/or Torres Strait Islander women. Probability estimates at: marriage=Married or de facto, state=NSW, patient class=Public, year=2014, mothers age=25 years, BMI=25.2 26 kg/m^2^, parity=2, antenatal visits=8, gestational age at first visit=12 weeks, diabetes status=No diabetes, hypertension=No hypertension.
